# Factors influencing adults' willingness to be screened for dementia risk

**DOI:** 10.1002/alz.091041

**Published:** 2025-01-03

**Authors:** Alexandra M Reed, Andrew Hooyman, Nicole M Lee, Elizabeth B. Fauth, Jill W Love, Sydney Y Schaefer

**Affiliations:** ^1^ Arizona State University, Tempe, AZ USA; ^2^ Arizona State University ‐ West Campus, Glendale, AZ USA; ^3^ Utah State University, Logan, UT USA; ^4^ Neurosessments LLC, Tempe, AZ USA

## Abstract

**Background:**

The Alzheimer’s Association recommends early screening of Alzheimer’s disease and related dementias(ADRD), urging the development of biomarkers and other tools for early risk/detection. However, the general public’s willingness to be tested for early ADRD must be considered, particularly among minoritized populations. For example, Black older adults within the United States (U.S.) are twice as likely to develop ADRD compared to White non‐Hispanic older adults, but have lower rates of engagement/trust in the medical system.

**Method:**

This study used Amazon MTurk to survey 572 U.S. adults on their willingness to take a (hypothetical) test for short‐term dementia risk. Mean participant age was 41.9±11.9. The sample was 47% Female, 77% non‐White, and 92% non‐Hispanic/Latino, with 37% having less than a bachelor’s degree. We also calculated an aggregated Area Deprivation Index (ADI) based on 5‐digit zip code to quantify a location’s relative advantage or disadvantage on a scale of 1‐100 compared to state and national percentiles, where lower ADI values represent greater area deprivation (mean±SE ADI = 47.65±24.51). All participants were non‐demented based on the Functional Assessment Questionnaire (score<9).

**Result:**

Although White and non‐White participants were both willing to test (∼77%), non‐White participants were approximately four times more likely to test only if their results were not shared with their medical provider/insurer. The desire to test outside of medical environments is further supported by the fact that the most common reason for taking the test was peace of mind (78%) rather than facilitating treatment plans, and that a positive test result would be most shared with family (62%) rather than with doctors right away. Older age was also associated with increased willingness to test, while ethnicity and socioeconomic status were not.

**Conclusion:**

Historical contexts and marginalization of minoritized groups may also impact willingness to screen for early ADRD risk/detection, which needs to be explored further. Building medical trust may take decades for some populations, thus supporting the need to develop options now for biomarkers or other ADRD‐risk screeners that are accessible and appropriate for culturally diverse groups, both within and outside of a medical context.

